# Late-onset Pompe’s disease in pediatrics: results from an Italian national survey on 38 patients and proposal of a targeted diagnostic algorithm

**DOI:** 10.1186/s13023-025-04063-x

**Published:** 2025-11-28

**Authors:** Marco Spada, Serena Gasperini, Massimiliano Filosto, Guja Astrea, Beatrice Bracci, Claudio Bruno, Alberto Burlina, Anna Cavallini, Daniela Concolino, Viola Crescitelli, Adele D’Amico, Federica Deodato, Carlo Dionisi-Vici, Maria Alice Donati, Simona Fecarotta, Rita Fischetto, Agata Fiumara, Francesca Furlan, Vincenza Gragnaniello, Damiano Mala, Monica Marica, Francesca Menni, Veronica Pagliardini, Claudia Panicucci, Giancarlo Parenti, Andrea Pession, Federica Ricci, Valentina Rovelli, Michele Sacchini, Filippo Maria Santorelli, Lucia Santoro, Maurizio Scarpa, Roberta Taurisano, Albina Tummolo, Francesco Porta

**Affiliations:** 1 Department of Pediatrics, University of Torino, Torino, Italy; 2 Department of Pediatrics, Fondazione IRCCS San Gerardo Dei Tintori, Monza, Italy; 3 Department of Clinical and Experimental Sciences, NeMO-Brescia Clinical Center for Neuromuscular Diseases, University of Brescia, Brescia, Italy; 4 Department of Developmental Neuroscience, IRCCS Fondazione Stella Maris, Pisa, Italy; 5 Centre of Translational and Experimental Myology, Department of Neuroscience, Rehabilitation, Ophthalmology, Genetics, Maternal and Child Health-DINOGMI, IRCCS Istituto Giannina Gaslini, University of Genoa, Genoa, Italy; 6 Division of Inherited Metabolic Diseases, Department of Women’s and Children’s Health, University of Padua, Padua, Italy; 7 IRCCS Fondazione Don C. Gnocchi, Milan, Italy; 8 Pediatric Unit, Department of Science of Health, Magna Graecia University of Catanzaro, Catanzaro, Italy; 9 Unit of Muscular and Neurodegenerative Disorders, Bambino Gesù Children’s Hospital, IRCCS, Rome, Italy; 10 Division of Metabolic Diseases and Hepatology, Bambino Gesù Children’s Hospital, IRCCS, Rome, Italy; 11 Metabolic Diseases and Neuromuscular Disorder, AOU Meyer-IRCCS, Firenze, Italy; 12 Department of Translational Medical Sciences, Federico II University of Naples, Naples, Italy; 13 Metabolic and Genetic Diseases, Policlinico di Bari Stabilimento Pediatrico Giovanni XXIII, Bari, Italy; 14 Regional Referral Centre for Metabolic Diseases, AOU Policlinico “G. Rodolico-San Marco”, University of Catania, Catania, Italy; 15 Fondazione IRCCS Ca’ Granda Ospedale Maggiore Policlinico, Regional Clinical Center for Expanded Newborn Screening, Milan, Italy; 16 Clinica Pediatrica e Malattie Rare, Brotzu, Cagliari, Italy; 17 Pediatric Unit, IRCCS Azienda Ospedaliero-Universitaria di Bologna, 40138 Bologna, Italy; 18 Child and Adolescent Neuropsychiatry, University of Turin, Turin, Italy; 19 Clinical Department of Pediatrics, San Paolo Hospital, ASST Santi Paolo e Carlo, University of Milan, Milan, Italy; 20 Division of Pediatrics, Department of Clinical Sciences, Azienda Ospedaliero Universitaria delle Marche, Presidio Salesi, Ancona, Italy; 21 Regional Coordinator Centre for Rare Diseases, University Hospital of Udine, 33100 Udine, Italy

**Keywords:** Pompe’s disease, Glycogen storage disease type II, Acid α-glucosidase, Late-onset Pompe’s disease

## Abstract

**Background:**

Late-onset Pompe’s disease (LOPD) is a progressive treatable metabolic myopathy due to partial acid α-glucosidase (GAA) deficiency, with potential onset during the pediatric age. To date, Pompe’s disease is not widely included in newborn screening panels, so that clinical suspect remains essential for timely diagnosis and management. Clinical identification of LOPD was shown to be challenging in adult patients, whereas data in children and adolescents are scanty. We conducted an Italian nationwide multicentric survey in order to delineate the characteristics of LOPD in the pediatric population. This prompted us to propose a diagnostic algorithm to facilitate the identification of LOPD in pediatrics.

**Results:**

The survey provided information on 38 Italian pediatric patients with a definite biochemical and molecular diagnosis of LOPD firstly suspected based on clinical approach. Nineteen patients (50%) reached medical attention because of clinical symptoms of LOPD (79% within 3 years of age; 21% at 3–18 years of age) and 19 (50%) because of incidental finding of asymptomatic hyperCKemia. All the 38 LOPD patients showed hyperCKemia (56%: range 500–1000 U/l; 18%; range 250–500 U/l; 18% range 1000–2000 U/l; 8% >2000 U/l), almost invariably accompained by concomitant hypertransaminasemia (91%). Main clinical symptoms before 3 years of age were inability to (1) sit at 8 months, (2) walk indipendently at 18 months, and (3) climb stairs at 30 months. Later pediatric presentations (3–18 years of age) were limitation to (1) get up from supine position, (2) perform sport activity, and (3) run. In symptomatic patients, diagnostic latency (i.e. the time interval between clinical onset and diagnosis of LOPD) ranged from < 1 year (58%) to 2–12 years (42%).

**Conclusions:**

Clinical diagnosis of LOPD in pediatrics is challenging in spite of its frequent presentation within 3 years of age. A selective screening by measuring GAA activity on dried blood spot in the two main clinical diagnostic contexts of LOPD in pediatrics – namely (1) age-related myopathic symptoms or (2) asymptomatic hyperCKemia (and hypertransaminasemia) – will likely ensure diagnostic anticipation for those patients not screened for Pompe’s disease in the neonatal period.

## Introduction

Pompe’s disease (OMIM 232300, glycogen storage disease type II) is an autosomal recessive lysosomal storage disorder due to over 400 mutations in the GAA gene, encoding for the acid α-glucosidase (GAA) enzyme [1]. Deficiency of GAA leads to lysosomal glycogen accumulation in all tissues, with most notable clinical effects in heart and skeletal muscles. Complete or near-complete GAA deficiency leads to classical Pompe’s disease, characterized by foetal involvement, life-threathening neonatal hypertrophic cardiomyopathy, and severe hypotonia. Reduced but residual GAA activity, on the other hand, is associated to late-onset variants of Pompe’s disease (LOPD), with highly variable clinical presentation during pediatric, adolescent or adult age [2]. As Pompe’s disease can be effectively treated by enzyme replacement therapy, a timely diagnosis is essential for its optimal management [3–6].

In different studies, newborn screening was effective in detecting both classic Pompe’s disease and LOPD, with a cumulative incidence of ranging from 1:8000 to 1:23000 [7–11]. Currently, however, Pompe’s disease is not widely included in newborn screening panels in Europe, so that the identification of patients still relies on clinical diagnosis. This approach, however, may be challenging especially for the diagnosis of LOPD, due to its variable and/or subtle clinical presentation. Actually, in previous studies, we showed that patients with idiopathic paucisymptomatic hyperCKemia, ~2% were actually affected by LOPD, consistent with a large clinical under-recognition of this condition both in pediatrics and adulthood [12, 13].

As for the pediatric population, on the other hand, information on the prevalence and clinical features of LOPD is scanty. Here, we report the results of the first Italian nationwide multicentric survey on pediatric LOPD, aimed to delineate clinical and biochemical characteristics of children and adolescents clinically diagnosed with this disease.

## Methods

Between January and March 2022, a nationwide survey was conducted in the 18 Italian metabolic Centers following children and adolescents (< 18 years) with LOPD. In all considered patients, GAA deficiency was confirmed both at enzymatic and molecular level. In particular, GAA activity on dried blood spots and on leukocytes ranged 0.4–0.9 nmol/ml/h (normal values > 3 nmol/ml/h) and 1.9–3.2 nmol/mg/h (normal values 14.8–27.2 nmol/mg/h), respectively. The common c.32-13T > C LOPD mutation in the GAA gene was harbored by 89% of patients; remaining patients harbored different variants, namely c.1927G > A, c.1064T > C, c.1655T > C, and c.1336G > C. Only cases of LOPD diagnosed on the basis of clinical and/or biochemical presentation were included; patients identified through newborn screening programs were excluded. A questionnaire was developed by a panel of expert physicians in order to retrospectively collect data on pediatric patients with LOPD. The survey was limited to data that could be reliably collected from medical charts. The survey investigated the history of LOPD, including age at onset, age at diagnosis, gender, and main clinical and biochemical characteristics. All data were presented as aggregate. Clinical signs and symptoms at presentation were categorized according to age (0–3 years or 3–18 years). Based on the results of the survey, the expert panel developed an evidence-based diagnostic algorithm for the diagnosis of LOPD in routine pediatric clinical practice. The study was conducted in accordance with the Declaration of Helsinki.

## Results

**Fig. 1:**
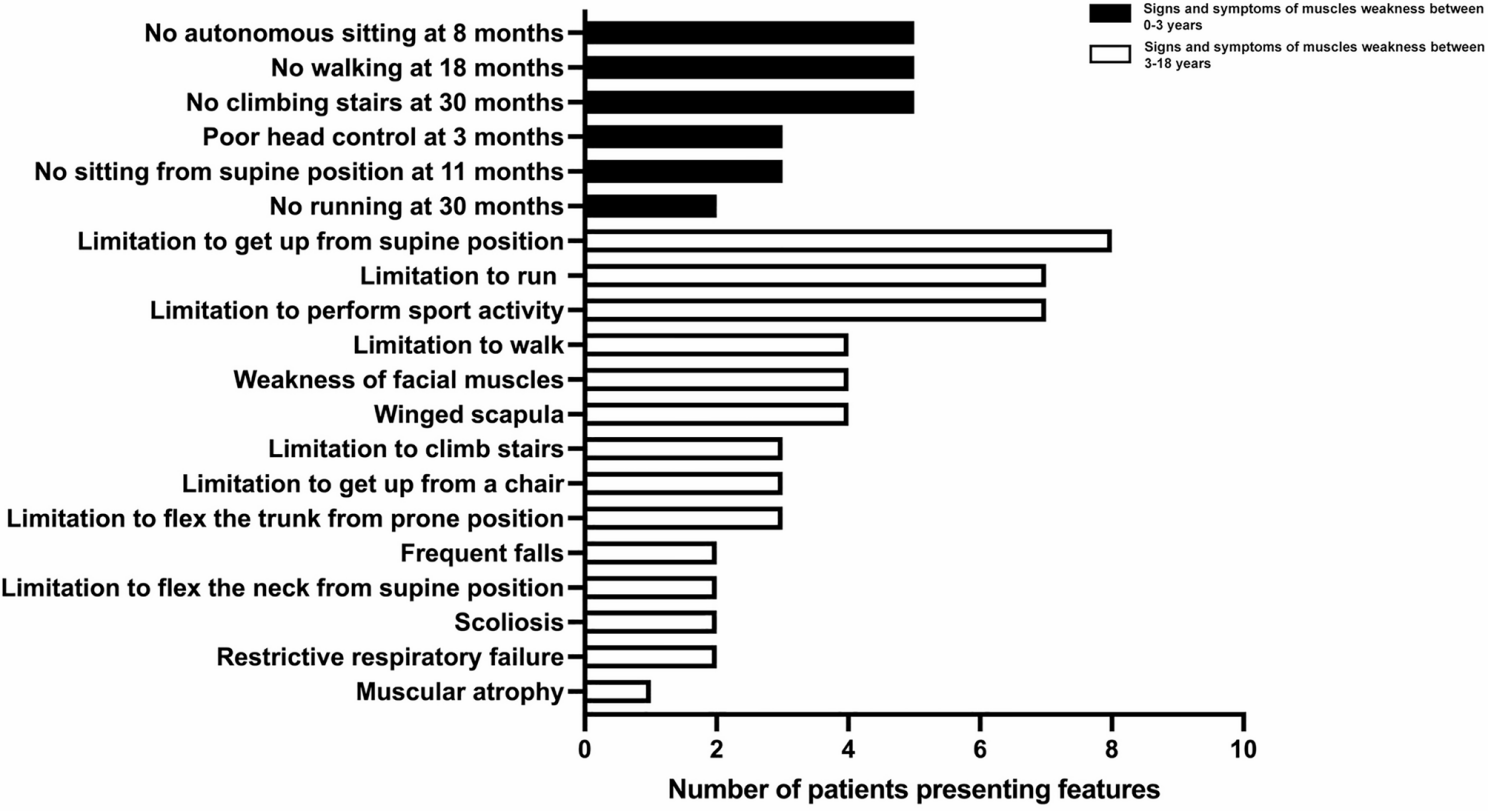
Clinical signs/symptoms of the 19 symptomatic patients with late-onset Pompe’s disease (LOPD) according to age (0–3 years, black bars; 3–18 years, white bars)

The survey provided information on the 38 known Italian pediatric patients with LOPD at March 2022 (not including those detected through newborn screening pilot studies). Nineteen patients (50%) reached medical attention because of clinical signs/symptoms of muscular weakness, whereas 19 patients (50%) were referred because of incidental evidence of persistent asymptomatic hyperCKemia. Clinical and biochemical details of the whole survey population are reported in Tables 1 and 2, respectively, with no difference between males and females. Among the 19 symptomatic patients, 15 (79%) had clinical onset within 3 years of age. A full picture of clinical signs/symptoms and their age-related prevalence is presented in Fig. 1. In particular, in the age range 0–3 years, the most common clinical features were (1) inability to sit autonomously at 8 months, (2) inability to walk independently at 18 months, and (3) inability to climb stairs at 30 months. In the age range 3–18 years, the clinical signs were (1) limitation to get up from supine position, (2) limitation to perform sport activity, and (3) limitation to run. In the symptomatic cohort, the time interval between clinical onset and a defined diagnosis of LOPD (i.e. diagnostic latency) was < 1 year in 11/19 patients (58%) and 2–12 years in 8/19 patients (42%). In particular, the 2–12 years diagnostic delay was in spite of clinical onset of LOPD in the age range 0–3 years in 7/8 patients (88%). In all symptomatic patients the serum CK level was high and serum transaminases level was high in 14/16 patients (test not available in 3 patients) (88%).

**Table 1:** Clinical characteristics of 38 Italian pediatric patients with late-onset pompe’s disease (LOPD)

Patients’ characteristics	Number of patients/population (%)
**Gender**	
Male	24/38 (63%)
Female	14/38 (37%)
**Reason of medical referral**	
Signs/symptoms of muscular weakness	19/38 (50%)
Asymptomatic incidental hyperCKemia	19/38 (50%)
**Age at diagnosis**	
0–3 years	10/38 (26%)
3–18 years	28/38 (74%)
**Biochemical characteristics at diagnosis**	
HyperCKemia	38/38 (100%)
Hypertransaminasemia	32/35 (91%)

**Table 2:** Biochemical characteristics at diagnosis of 38 Italian pediatric patients with late-onset pompe’s disease (LOPD)

Patients’ biochemical characteristics	Number of patients/population (%)
**CK range**	
Normal values	0/38 (0%)
250–500 U/L	7/38 (18%)
500–1000 U/L	21/38 (56%)
1000–2000 U/L	7/38 (18%)
> 2000 U/L	3/38 (8%)
**Transaminases range**	
Normal values	3/35 (9%)
70–150 U/L	17/35 (48%)
150–500 U/L	13/35 (37%)
500–1000 U/L	2/35 (6%)

The 19 asymptomatic patients with LOPD reached medical attention because of incidental evidence of persistent hyperCKemia at routinary medical analyses, almost invariably accompanied by concomitant mild hypertransaminasemia (18/19 patients, 95%).

## Discussion

LOPD is a progressive myopathy due to partial GAA deficiency with potential onset in the pediatric age. Clinical presentation and biochemical characteristics of LOPD in pediatrics were only anecdotally investigated [14–16]. Based on available newborn screening data in Italy on over 200,000 newborns, the incidence of LOPD is 1:25000 [11]. In the context of the Italian pediatric population (9000000 of people ranging 0–18 years, data from the National Institute of Statistics), the epidemiological picture from newborn screening data might be consistent with the actual presence of ~ 350 pediatric subjects with LOPD in Italy. The comparison of this extrapolated epidemiology with the real data from our nationwide survey evidences that the current known Italian pediatric population with LOPD (38 patients) virtually accounts only for ~ 10% of the expected subjects with LOPD in the 0–18 years age range, suggesting a low clinical diagnostic rate for this disease.

Moreover, our nationwide survey provided a large body of additional information on pediatric LOPD. First, we observed a dichotomous context of diagnosis of LOPD, with half patients (19/38) diagnosed based on clinical symptoms and half (19/38) based on the incidental finding of hyperCKemia in pre-symptomatic patients. Second, in the Italian experience, all pediatric patients with LOPD (symptomatic and presymptomatic) exhibited persistent hyperCKemia, often accompanied by mild hypertransaminasemia. Third, even in early symptomatic patients (i.e. clinical onset before of 3 years), the identification of LOPD may be challenging also for the timing of treatment, as demonstrated by a 6-year diagnostic latency from clinical onset [17]. This figure, indeed, is in agreement with the above-mentioned low clinical diagnostic rate of LOPD in the Italian pediatric population [18].

**Fig. 2:**
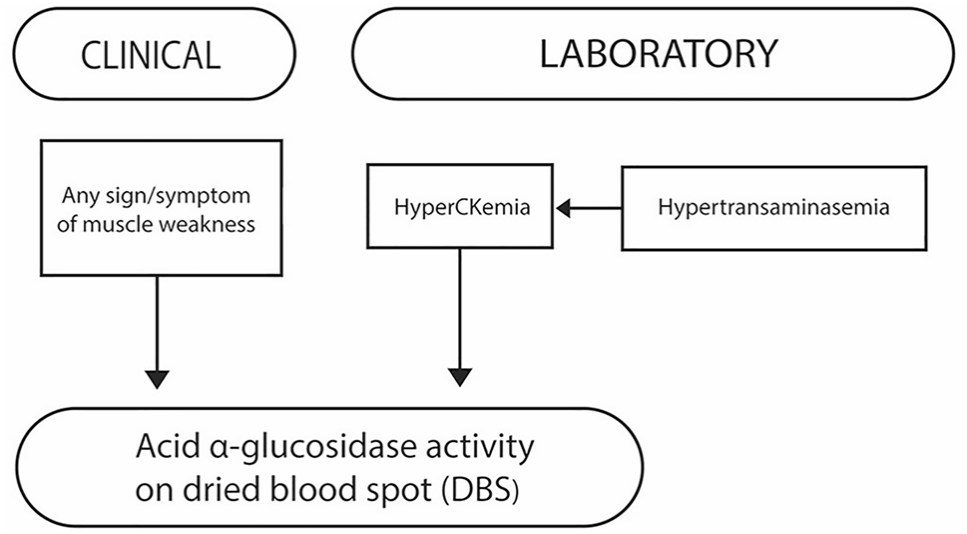
A diagnostic algorithm to identify late-onset Pompe’s disease (LOPD) in routine pediatric clinical practice

Based on these results and observations, a simplified diagnostic algorithm was elaborated in order to improve the clinical diagnostic rate of LOPD in routine clinical pediatrics (Fig. 2). As shown in the algorithm, three main clinical scenarios should systematically address the suspicion of LOPD in routine pediatric practice, namely (1) any sign or symptom of muscular weakness, (2) any evidence of persistent hyperCKemia, and (3) any hypertransaminasemia. In particular, both muscular symptoms or hyperCKemia should immediately address the determination of GAA activity in dried blood spot, whereas any hypertransaminasemia should mandatorily be accompanied by searching for hyperCKemia and, in the case, address again GAA activity measurement on dried blood spot.

In order to overcome the potential limitation of this study due to the single-country selected population, the proposed algorithm takes also into account the evidence from the literature that patients with LOPD from different populations can present with isolated clinical symptoms with steadily normal CK levels, so that its application might be likely suitable worldwide [19, 20].

Hopefully, the systematic application of the proposed clinical diagnostic algorithm as a selective screening in pediatrics will facilitate the biochemical diagnosis and molecular confirmation of LOPD in affected but still undiagnosed subjects, allowing the optimization of their clinical management and genetic counselling until the wide inclusion of Pompe’s disease in newborn screening panels.

## Data Availability

The data that support the findings of this study are not openly available due to reasons of sensitivity and are available from the corresponding author upon reasonable request. URL: https://we.tl/t-bYxJILso8s
